# Decaffeinated Green Tea Extract Does Not Elicit Hepatotoxic Effects and Modulates the Gut Microbiome in Lean B6C3F_1_ Mice

**DOI:** 10.3390/nu11040776

**Published:** 2019-04-03

**Authors:** Bill J. Gurley, Isabelle R. Miousse, Intawat Nookaew, Laura E. Ewing, Charles M. Skinner, Piroon Jenjaroenpun, Thidathip Wongsurawat, Stefanie Kennon-McGill, Bharathi Avula, Ji-Yeong Bae, Mitchell R. McGill, David Ussery, Ikhlas A. Khan, Igor Koturbash

**Affiliations:** 1 Department of Pharmaceutical Sciences, University of Arkansas for Medical Sciences, Little Rock, AR 72205-7199, USA; GurleyBillyJ@uams.edu; 2 Center for Dietary Supplements Research, University of Arkansas for Medical Sciences, Little Rock, AR 72205-7199, USA; CMSkinner@uams.edu (C.M.S.); MMcgill@uams.edu (M.R.M.); 3 Department of Environmental and Occupational Health, University of Arkansas for Medical Sciences, Little Rock, AR 72205-7199, USA; IRacinemiousse@uams.edu (I.R.M.); LEEwing@uams.edu (L.E.E.); SKennonmcgill@uams.edu (S.K.-M.); 4 Department of Biochemistry and Molecular Biology, University of Arkansas for Medical Sciences, Little Rock, AR 72205-7199, USA; 5 Department of Biomedical Informatics, University of Arkansas for Medical Sciences, Little Rock, AR 72205-7199, USA; INookaew@uams.edu (I.N.); PJenjaroenpun@uams.edu (P.J.); TWongsurawat@uams.edu (T.W.); DWUssery@uams.edu (D.U.); 6 Department of Pharmacology and Toxicology, University of Arkansas for Medical Sciences, Little Rock, AR 72205-7199, USA; 7 National Center for Natural Product Research, School of Pharmacy, The University of Mississippi, University, MS 38677, USA; bavula@olemiss.edu (B.A.); jbae7@olemiss.edu (J.-Y.B.); ikhan@olemiss.edu (I.A.K.)

**Keywords:** catechins, green tea extract, herbal dietary supplements, hepatotoxicity, microbiome

## Abstract

The main purpose of this study was to investigate the hepatotoxic potential and effects on the gut microbiome of decaffeinated green tea extract (dGTE) in lean B6C3F_1_ mice. Gavaging dGTE over a range of 1X–10X mouse equivalent doses (MED) for up to two weeks did not elicit significant histomorphological, physiological, biochemical or molecular alterations in mouse livers. At the same time, administration of dGTE at MED comparable to those consumed by humans resulted in significant modulation of gut microflora, with increases in *Akkermansia* sp. being most pronounced. Results of this study demonstrate that administration of relevant-to-human-consumption MED of dGTE to non-fasting mice does not lead to hepatotoxicity. Furthermore, dGTE administered to lean mice, caused changes in gut microflora comparable to those observed in obese mice. This study provides further insight into the previously reported weight management properties of dGTE; however, future studies are needed to fully evaluate and understand this effect.

## 1. Introduction

The importance of dietary polyphenols for systemic health benefits is becoming increasingly recognized. Green tea, a major source of catechin polyphenols, is the second most popular beverage in the world and extracts of green tea are common ingredients in many dietary supplements. Major green tea extract (GTE) catechins include epicatechin (EC), epicatechin gallate (ECG), epigallocatechin (EGC) and epigallocatechine gallate (EGCG), where the latter constitutes 50-80% of total catechins [1,2]. Catechins are reported to exert a number of positive effects on human health, including antioxidant, antibacterial and anti-inflammatory activities as well as reduced risks for cancer and cardiovascular disease [3,4,5,6]. Furthermore, the association of green tea or GTE consumption with weight loss and weight management, has further attracted interest to studies on catechins [7,8]. While these claims are based mostly upon the results of animal studies or equivocal clinical trial findings, the popularity of GTE and GTE-containing herbal dietary supplements (HDS) continues to grow. At the same time, GTE and its various catechin components (mainly–EGCG) are linked to a number of hepatotoxicity cases [9,10,11,12,13,14,15]. This hepatotoxicity has been confirmed experimentally and was shown to be further exacerbated by fasting conditions [16,17,18,19]. Therefore, the first aim of this study was to investigate potential hepatotoxicity and associated mechanisms of decaffeinated GTE (dGTE) in non-fasting mice.

The potentially beneficial effects associated with GTE and their mechanisms remain poorly understood. It has been demonstrated that short-term ingestion of GTE increases energy expenditure and promotes weight loss among lean and overweight volunteers but the long-term effects of GTE on energy expenditure were less conclusive [20,21,22,23,24,25]. Other hypotheses include GTE-mediated effects on sympathetic nervous system activity and promotion of fat oxidation [8]. Furthermore, a number of in vitro studies have indicated that EGCG inhibits adipocyte differentiation and proliferation while inducing adipocyte apoptosis [26,27,28]. However, it must be recognized that most in vitro studies have utilized EGCG concentrations (50–400 µM) much greater than that typically observed in humans (up to 1 µM) following GTE ingestion [29]. Furthermore, it is become increasingly recognized that intestinal absorption of catechins is at best nominal with less than 30% of ingested green tea polyphenols reaching the systemic circulation [30,31,32,33,34]. Poor absorption coupled with extensive first-pass metabolism likely explains the poor tissue accumulation of catechins following oral ingestion [35,36]. Therefore, the purported health benefits of GTE are not readily attributable to circulating levels of catechins.

Substantial levels of unabsorbed catechins, mainly EGCG, have been shown to reach the proximal and distal colon [37,38]. To what extent gut microbial metabolism plays a role in mediating GTE’s health benefits remains to be determined. However, accumulating evidence indicates that GTE can modulate the gut microbiome in both experimental models and in humans [39,40,41,42]. Therefore, it has been proposed that GTE’s health benefits may be linked to the effects catechins exert on particular bacterial species in the gut. Recent studies report similar patterns in the effects GTE causes on the gut microbiome in both experimental models and in human subjects. Those patterns are characterized by higher Shannon and Simpson microbiome diversities, increases in abundance of *Bacteroidetes* concomitant with deceases in *Firmicutes* at the phyla level and increases in *Prevotellaceae* and *Bacteroidaceae* paralleled by decreases in *Eubacteriaceae*, *Lachnospiraceae*, *Ruminococcaceae* and *Clostridiaceae* at the family level [39,40,41]. These studies, however, were performed on obese individuals or obese/fed high-fat diet mice; however, the effects of GTE on the gut microbiome associated with the lean phenotype remain unknown. Therefore, the second aim of this study was to investigate the effects of dGTE on the gut microbiome as a result of short-term ingestion in lean B6C3F_1_ mice.

## 2. Materials and Methods

### 2.1. Decaffeinated Green Tea Extract (dGTE)

The studied product was a standardized dGTE manufactured by Nature’s Way (Green Bay, WI, USA; lot # 20055697, expiration 11/30/18). The gavage solution was prepared by extracting the contents of 10 capsules with 10 mL of distilled water (pH = 5.3) in 20 mL round bottom, glass screw cap tubes via rotation (12 revolutions per minute) for 24 h. Tubes were then centrifuged at 10,000 rpm for 1 h, the supernatant was collected and two 1 mL aliquots were analyzed by the University of Mississippi’s National Center for Natural Products Research for analysis (NCNPR).

dGTE was characterized for phytochemical content using validated analytical methods incorporating ultra-high performance liquid chromatography (UPLC) coupled with photodiode array (PDA) and mass spectrometry (MS) detection previously developed for the quantitative analysis of caffeine, theobromine and individual catechins (i.e., catechin, epicatechin, epicatechin gallate, epigallocatechin gallate) in *Camellia sinensis* leaves and GTE-containing products. Quantitative analysis was performed using a Waters Acquity UPLC^TM^ H-class system (Waters Corp., Milford, MA, USA) including a quaternary solvent manager, sample manager, column compartment and PDA (Waters Acquity model code UPD) connected to a Waters Empower 2 data station. Separations were achieved within 15 min using a Waters C_18_ column. The injection volume was 2 µL and the PDA wavelength was 230 nm. The effluent from the LC column was directed into an electrospray ionization (ESI) probe. Compounds were confirmed under both positive and negative ionization modes.

### 2.2. Animals

Male B6C3F_1_/J mice, 8 weeks of age, were purchased from Jackson Laboratories (Bar Harbor, ME) and were housed at the UAMS Division of Laboratory Animal Medicine facility. B6C3F_1_/J mice are characterized by an average sensitivity to hepatotoxicants and are widely used by both the U.S. Food and Drug Administration (FDA) and industry to investigate the potential for xenobiotics to produce hepatotoxicity. Male mice were used on account of previous reports indicating a higher sensitivity to GTE-induced toxicity in male animals [17]. Animals were given one week to acclimate before the initiation of studies. Animal experiments were conducted in two stages. In the first stage, mice were gavaged with a single dose of either 1X, 3X or 10X mouse equivalent doses (MED) of dGTE with the subsequent tissue harvest at 24 h. This stage was performed in order to address the potential for acute toxicity of dGTE. During the second stage, mice were gavaged with dGTE for two weeks (Mon-Fri). The duration of this stage was chosen to investigate dGTE’s sub-acute toxicity. To avoid potential fasting-exacerbated toxicity, food and water were provided *ad libitum*. Animal body weights were measured and recorded twice a week. All procedures were approved by the UAMS Institutional Animal Care and Use Committee at UAMS (protocol number: AUP #3701).

### 2.3. Dosage Information/Dosage Regimen

Allometric scaling for mouse equivalent doses for dGTE was determined per the recommendation of Wojcikowski and Gobe [43] which, in turn, is based upon the FDA Industry Guidance for Estimating the Maximum Safe Starting Dose in Initial Clinical Trials for Therapeutics in Adult Volunteers [44].

According to the label, each capsule 250 mg of dGTE was standardized to 95% polyphenols (75% catechins). Label recommended dose was “2 capsules daily, preferably with food.” The human dose of catechins was calculated to be 375 mg catechins/70 kg = 5.36 mg/kg. MED of dGTE was calculated as 5.36 mg/kg × 12.3 = 65.9 mg/kg, where 12.3 is the scaling factor commonly used for mice weighing between 11–34 g. Concentration of total catechins per mL for the Nature’s Way extraction solution as determined by NCNPR was 723.5 mg/mL. Therefore, for the 1X MED, the quantity of catechins administered was 65.9 mg/kg × 0.0235 kg (average mouse weight in our study) = 1.5 mg total catechins delivered in 300 µL of gavage solution. Consequently, 3X MED = 4.5 mg total catechins and 10X MED = 15 mg total catechins.

All extract supernatants were kept in the refrigerator and gavage doses were prepared fresh each day. After 40 days, a reanalysis of the catechin content of the supernatants was performed and the total catechin concentration was 92% of the original quantity.

### 2.4. Blood Sampling and Clinical Biochemistry

To measure the effects of dGTE on the panel of enzymes characteristic for liver injury, blood was collected at the end of each experimental stage. Blood was collected under isoflurane anesthesia from the retroorbital plexus. Tubes were kept on ice and centrifuged at 10,000 rpm for 20 min; serum samples were then immediately aliquoted and delivered to Arkansas Livestock and Poultry Commission Veterinary Diagnostic Laboratory (Little Rock, AR, USA) where the samples were processed same day.

### 2.5. Histopathological Assessment

Livers were excised, and a 1 mm section was obtained from the left lateral lobe and another from the right medial lobe. The sections were fixed in 4% formalin for 24 h, then briefly rinsed in PBS and stored in 70% ethanol for 24 h. Livers were then processed at the UAMS Pathology Core Facility, stained with hematoxylin eosin and shipped to the Heartland Veterinary Pathology Services, PLLC (Edmond, OK) where they were assessed by a board-certified veterinary pathologist in a blind fashion.

For histologic evaluation purposes, each liver was represented by two sections obtained from different lobes. Each section was initially evaluated at magnifications of 4 × 0 and 100X. The sections were then evaluated at 200X and 400X to better determine if significant changes were present and to check for the presence of mitotic figures and apoptotic bodies.

### 2.6. Glutathione Analysis

Glutathione was measured using a modified Tietze assay [45]. Briefly, liver tissue was homogenized in 3% sulfosalicylic acid. One aliquot was diluted in N-ethylmaleimide (NEM) to mask reduced glutathione (GSH) to facilitate measurement of oxidized glutathione (GSSG), while another was diluted in 0.1 M HCl for measurement of total (GSH+GSSG) glutathione. After removal of NEM by solid phase extraction with a C18 column, glutathione was measured in both aliquots using a colorimetric glutathione reductase cycling detection method [45].

### 2.7. Gene Expression Array

Total RNA was extracted from flash frozen liver tissue using the RNeasy Mini Kit (Qiagen, Germantown, MD, USA). Following purification, 1000 ng were reverse transcribed with the High Capacity cDNA Reverse Transcription Kit (ThermoFisher, Waltham, MA, USA). The cDNA was diluted to 5 ng/µL and 105 µL was mixed with an equal volume of 2X TaqMan^®^ Fast Advanced Master Mix. For real-time PCR, 100 µL of the mix was applied to each of two channels on a TaqMan Low Density Hepatotoxicity Array (TLDA) (Supporting Information Table S1) (ThermoFisher, Waltham, MA, USA). Four biological samples were loaded on each array with five samples per each group analyzed. Analysis was performed using the ExpressionSuite Software v1.1 (ThermoFisher, Waltham, MA, USA).

### 2.8. Analysis of the Gut Microbiome

Fecal samples from individual mice were placed into collection tubes containing a nucleic acid stabilizer (Zymo Research, Irvine, CA, USA). Bacterial DNA extraction was performed using ZymoBIOMICS DNA Kits (Zymo Research). In total, 400 ng of each sample was used for tagmentation and library preparation, as directed by manufacturer’s protocol of KAPA HyperPlus Kit (Roche, Madison, WI, USA). Then, each library was purified using AMPure XP bead (Beckman Coulter, Indianapolis, IN, USA). Normalized libraries were pooled and pair-end sequencing using the Illumina NextSeq 500 platform to obtain 150 bp paired-end reads was performed.

Raw Illumina fastq files were preprocessed to ensure that only the high-quality reads would be used for further bioinformatics analysis; adapter trimming and quality filtering were performed using Trimmomatic software version 0.36 with default parameters [46]. High quality fastqs were further used as the inputs for reference taxonomic classification and quantification using Centrigue version 1.0.4 with default parameters to generate species profile [47]. Profiles were then visualized on a taxonomic hierarchy using *Pavian* package for comparison purposes. The high quality reads were used for *de novo* assembly binning to construct high quality metagenomic gene profiling using the metaWRAP pipeline—a flexible pipeline for genome-resolved metagenomic data analysis with default parameters except using the mouse genome (mm10) to account for host contamination. Non-redundance gene sets were constructed as per Foong et al. from the obtained ORFs of the samples using Usearch fast clustering with identity cutoff of 95% and overlap length of 90% [48]. The constructed non-redundance gene sets were then translated into amino acid sequences for KEGG pathway annotation using ghostKOALA pipeline [48,49]. Differential abundance analysis of taxonomic and gene profiles were performed from the count data using DESeq2 package [50]. The adjusted *p*-values were then used for KEGG pathway enrichment analysis using piano package [51]. Pathways that had enrichment *p*-value of < 0.001 were selected to plot heatmaps. Raw sequence reads have been uploaded to NCBI, accession ID: PRJNA523806.

### 2.9. Statistical Analysis

All statistical analyses were performed with the GraphPad Prism 6 software (GraphPad Software. San Diego, CA, USA). Treatment groups were compared with their respective untreated group using ANOVA followed by Tukey’s multiple comparison test. In cases where the data was not normally distributed, a Kruskal-Wallis test followed by a Dunn’s multiple comparisons test was used instead.

## 3. Results

### 3.1. Phytochemical Characterization of Dgte Utilized in the Study

Phytochemical characterization of utilized dGTE is presented in Table 1. The catechin composition of the characterized product was comparable to the catechin composition in the product used in other animal studies with no more than 10% difference for each particular catechin ingredient.

### 3.2. Studies on Acute dGTE Toxicity

**Figure 1:**
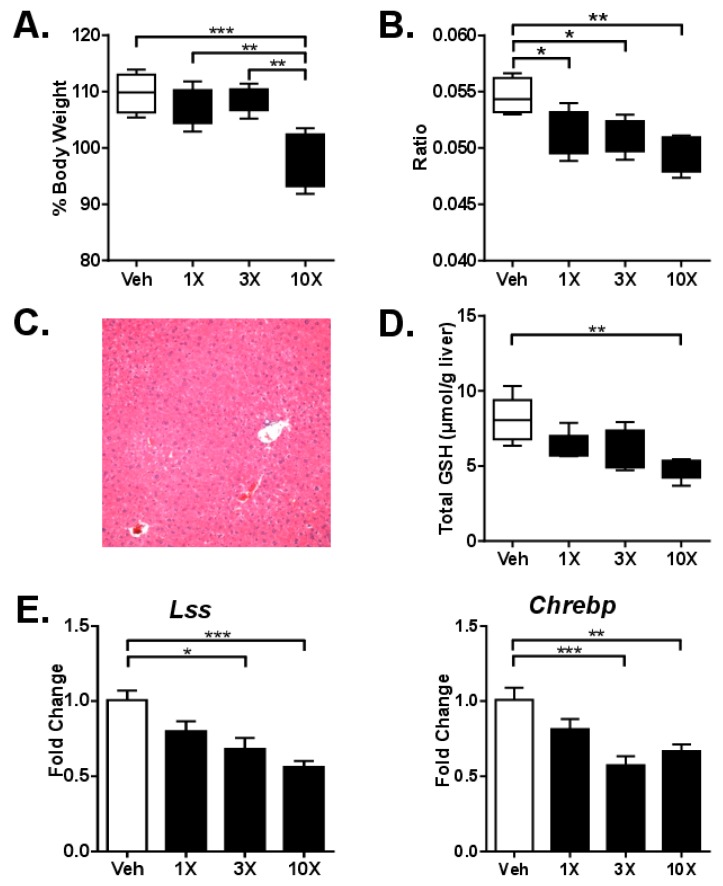
Analysis of dGTE acute toxicity. Body weights (**A**) and liver-to-body weight ratio (**B**). Photomicrograph of intact mouse liver after a single gavage with 10X mouse equivalent dose (MED) of dGTE (**C**). Total glutathione (**D**). mRNA levels of *Lss* and *Chrebp* genes (E). * *p* < 0.05, ** *p* < 0.01, *** *p* < 0.001; mean +/- SEM (*n* = 5 per group).

Acute toxicity was investigated 24 h after a single gavage of mice with either 1X, 3X or 10X MED of dGTE to determine if dGTE can cause hepatotoxicity in a fed state. Significant decreases in body weight were observed in mice gavaged with 10X MED (12%, *p* < 0.001) (Figure 1A). The liver-to-body weight ratio was slightly but significantly decreased in all experimental groups (Figure 1B). Moderate changes in the organ-to-body weight ratios were also observed in the heart but not in the kidney (Supporting Information Figure S1A,B). No appreciable differences in cytoplasmic vacuolation, apoptotic or mitotic events, nor steatosis were observed in the livers of control versus experimental animals (Figure 1C).

Analysis of clinical biochemistry did not reveal any substantial changes in any of the evaluated parameters, besides the insignificant nearly two-fold increase in ALT and ~20% increase in AST after gavage with 1X MED (Table 2). To determine if dGTE had any effect on glutathione concentration or generation of reactive oxygen species (ROS) in the liver, we measured both total (GSH+GSSG) and oxidized (GSSG) glutathione. dGTE dose-dependently decreased hepatic GSH+GSSG content at 24 h, with ~40% (*p* < 0.05) depletion at 10X MED (Figure 1D). On the molecular level, only two genes out of 84 investigated were significantly deregulated—*Lss* and *Chrebp*. The expression of both genes was decreased; however, the extent of the changes was low (below 2-fold) (Figure 1E).

### 3.3. Studies on Sub-Acute dGTE Toxicity

**Figure 2:**
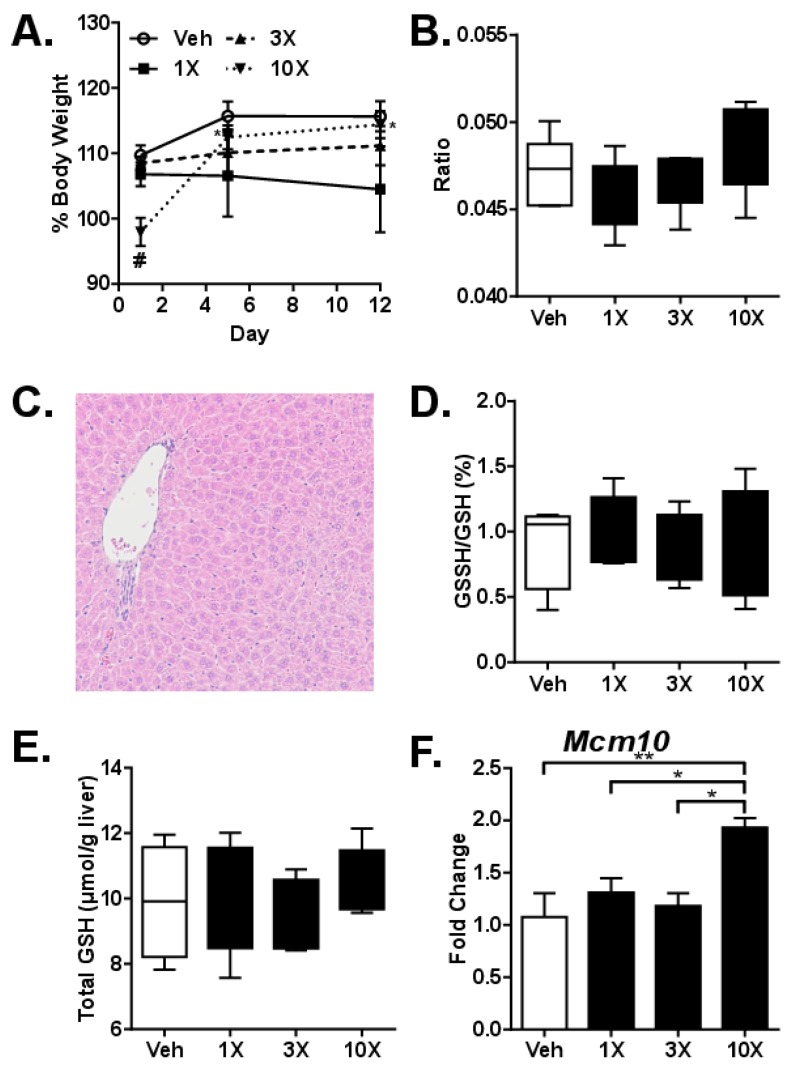
Analysis of dGTE sub-acute toxicity. Body weights ((**A**) # significant compared to vehicle, *significantly different from Day 1 within a dose group) and liver-to-body weight ratio (**B**). Photomicrograph of intact liver after gavaging mouse with 10X MED dGTE for 2 weeks (**C**). GSSH/GSH ratio (**D**), total glutathione (**E**) and mRNA levels of *Mcm10* gene (**F**). * *p* < 0.05, ** *p* < 0.01; ^#^ *p* < 0.05 compared to vehicle (**F**); mean +/- SEM (*n* = 5 per group).

Sub-acute toxicity was investigated after 2 weeks (Mon-Fri) of daily gavage with either 1X, 3X or 10X MED dGTE. A statistically significant decrease in body weight (8%, *p* = 0.012) was observed after gavaging mice with 1X MED dGTE compared to control mice at the end of the study (Figure 2A). No differences in body weight were observed after 3X and 10X MED dGTE. Gavaging with dGTE did not cause any changes in liver-to-body weight ratio (Figure 2B) as well as heart-to-body weight ratios (Supporting Information Figure S2A). A small increase in kidney-to-body weight ratio was observed in mice gavaged with 1X MED dGTE (Supporting Information Figure S2B).

Similar to the acute toxicity study, there were no histomorphological changes in the livers of experimental animals (Figure 2C). Furthermore, no changes were observed in the evaluated serum parameters except for the ~30% decrease in ALP in mice gavaged with 1X MED dGTE (Table 2). GSH+GSSG did not differ between groups after 2 weeks (Figure 2D), indicating compensatory GSH synthesis with prolonged exposure. Although the ratio of GSSG to GSH was significantly increased after GTE treatment at 24 h (data not shown), the absolute amount of GSSG was unchanged at both 24 h and 2 weeks (Figure 2E).

Gene expression analysis revealed only one (1) out of 84 genes significantly deregulated, *Mcm10*, increased expression of which was observed after administration of 10X MED dGTE (1.9-fold, *p* < 0.01) (Figure 2F).

### 3.4. Studies on the Gut Microbiome

Next, we sought to investigate whether or not orally administered dGTE affected the gut microbiome of lean mice. We selected 3X MED (equivalent of ~200 mg/kg/bw) since this is a dGTE dose analogous to that commonly consumed by humans [52].

**Figure 3:**
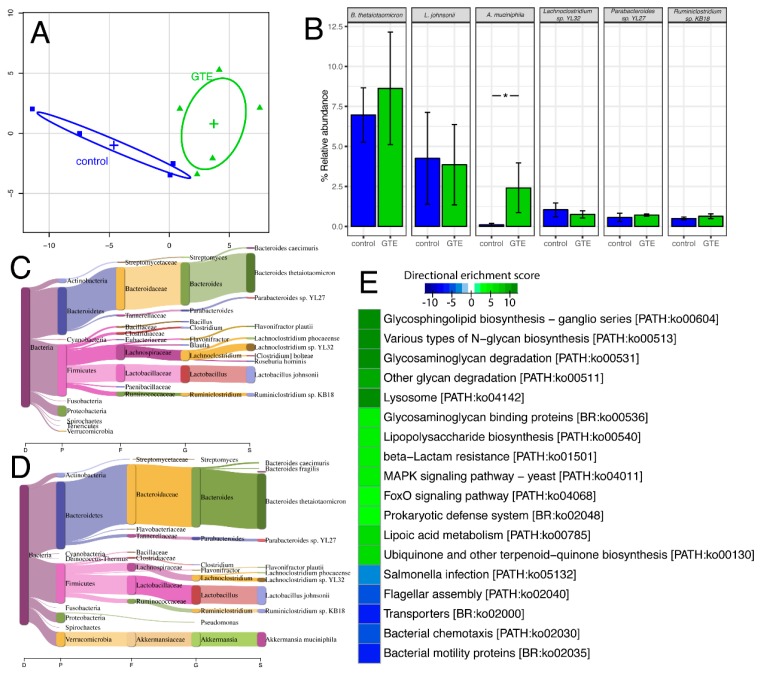
Shot gun metagenome analysis of dGTE (green) compare with control (blue). PCA plot of gut microbiome species abundance (**A**). Bag plots of high abundance gut bacteria (>5% relative abundance) in the study (**B**) * *p* < 0.05 compared to vehicle; mean +/- SEM (*n* = 5 per group). Sankey diagram for visualization of species abundance in a taxonomic tree of a sample control group (**C**) and dGTE (**D**). Heat map of directional enrichment score (−log10 enrichment *p*-value) for selected KEGG pathway (**E**).

We report that gavaging lean B6C3F_1_ mice with 3X MED dGTE for the period of two weeks, caused substantial perturbations in the mouse gut ecology. Taxonomic profiling using Centrifuge software identified a clear discrimination between the control and dGTE mice (Figure 3A). At the cut-off of 0.5% relative abundance, *B. thetaiotaomicron*, a common resident bacteria in a mouse gut, was the most abundant species, followed by *L. johnsonii*, *Akkermansia muciniphila*, *Lachnoclostridium sp. YL32*, *Parabacteroides sp. YL27 and Ruminoclostridium sp KB18* (Figure 3B). Administration of dGTE caused an increase in the *Bacteroidetes* to *Firmicutes* ratio (Figure 3C,D). Interestingly, only *A. muciniphila* abundance was dramatically increased in the dGTE group compared to control in the high abundance taxa (Figure 3C,D) with the most statistically significant adjusted *p*-value of 1.75e-7 (Supporting Information Figure S3, Table S2). Based on KEGG pathway analysis, increased abundance of genes associated with glycan degradation-related pathway in dGTE group compared to control was found (Figure 3E). As mucin is composed of different types of glycans, this correlates with the increased abundance of *A. muciniphila*, which is the main consumer of mucin in both human and animal gut [53]. On the other hand, decreased abundance of genes related with *Salmonella* infection, bacterial chemotaxis and bacterial mobility proteins in dGTE group was noted.

## 4. Discussion

To assess the hepatotoxic potential of dGTE, we utilized an integrative approach similar to our other recent studies for the safety assessment of multi-ingredient botanical dietary supplement formulations [54,55]. This approach considers analyses based upon: (1) the number of end-points characteristic for liver injury; (2) a dose range of 1X to 10X MED (65.9 to 659 mg/kg bw/day of dGTE for this study); and (3) single and repeated dosing studies. This allows for a fast and comprehensive investigation of phytochemical hepatotoxicity as well as provide insight into potential toxicological mechanisms.

Our findings are in agreement with previous pre-clinical and clinical studies on dGTE hepatotoxicity that reported a lack of liver injury at doses below ~750 mg/kg bw/day [13,16,52]. Despite administering dGTE at doses as high as 10X MED, no appreciable toxicological responses were observed in experimental mice. Specifically, gavaging mice with dGTE produced no histopathological abnormalities in the livers and no significant alterations were observed in clinical biochemistry parameters indicative of liver injury. Small decreases in total glutathione were observed in mice livers 24 h after a single administration of dGTE; however, these effects were short-lived and had disappeared by day 14. The fact that dGTE had no effect on GSSG at 1X and 3X MED argues against the idea that orally administered dGTE is an antioxidant and the observed depletion of GSH+GSSG after a bolus dose of dGTE may even increase the risk of oxidative stress. This finding underscores the necessity of validating in vitro data using in vivo models and warrants further in vivo studies to investigate the potential anti- and pro-oxidant effects of GTE [56,57].

Only very modest, dose-independent changes in gene expression were detected in the livers of dGTE-gavaged mice. Analysis of expression panels for genes involved in xenobiotic metabolism or hepatocellular responses to toxicants revealed only a small subset (<5%) that was significantly dysregulated. Importantly, the magnitude of responses in those genes was minimal, with only one gene, *Mcm10*, exceeding a 1.5-fold increase from control. Furthermore, reduced expression of *Lss* and *Chrebp* genes that are associated with cholesterol and glycogen metabolism in mice may suggest potentially beneficial health effects and warrant future studies. No dGTE-induced weight-loss was observed; however, this can be explained by the lean nature of the mice and the study’s short duration.

It must be noted that our study was performed under conditions that purposefully omitted other potential contributors to liver injury, such as genetics, fasting and caffeine [16,19,58]. GTE- or EGCG-induced liver injury is considered idiosyncratic by nature. While the mechanisms of this idiosyncrasy remain unknown, genetic components seem to play a significant, if not key, role [10,59]. Furthermore, in their elegant study using diversity outbred (DO) mice, Church and colleagues demonstrated that variations in select genomic loci may predispose to higher sensitivity to EGCG [19]. Therefore, our observed lack of dGTE-induced hepatotoxicity among inbred B6C3F_1_ mice, a strain characterized by average sensitivity to hepatotoxicants, is not surprising.

Previous research hints at a contribution of fasting in GTE/catechins-induced liver injury. For instance, in two classical studies with beagle dogs, fasted animals exhibited high sensitivity to orally administered EGCG, including mortality at doses of 400 mg/kg bw with No-Observed-Adverse-Effect-Level (NOAEL) observed at 40 mg EGCG/kg bw/day [16]. At the same time, the NOAEL in dogs that received food *ad libitum* was 460 mg/kg bw/day and could potentially have been higher, as this dose of EGCG was the highest used in the study [16]. In our study, the mice received food *ad libitum*, with a NOAEL of 659 mg/kg bw/day.

Furthermore, our study utilized dGTE, thereby precluding any contributory effects from caffeine [58]. Importantly, in many GTE-associated cases of hepatotoxicity, GTE was one but not the only, constituent of the formulation. For example, GTE was present in both Hydroxycut™ and X-elles™, two dietary supplement formulations linked to multiple cases of hepatotoxicity that were voluntarily withdrawn from the market [14]. Besides GTE, both of those formulations contained caffeine and a host of other botanical ingredients. Caffeine’s propensity to exacerbate the toxicity of other phytochemicals was recently recognized by the FDA, which banned the sale of pure caffeine powder and dietary supplements containing high caffeine content [60].

Finally, product adulteration with prescription medications (e.g., acetaminophen, amphetamines, etc.) or contamination with heavy metals, pesticides/herbicides or bacteria cannot be ruled as contributors to the hepatotoxicity of multi-ingredient, GTE-containing supplements [10,11]. Phytochemical characterization of the dGTE used in the present study revealed no evidence of adulteration, heavy metal or bacterial contamination.

Accumulating evidence indicates that catechin oral bioavailability is relatively low [13,15,16,17,18,34,35]. Our findings, together with a wealth of previously published data, suggest that any dGTE-derived health effects from catechins, likely stem from dGTE-mediated alterations in the distal gut microbiome and potential active metabolites generated therein, rather than from catechin absorption in the proximal intestine. Indeed, even minimal dietary interventions can substantially affect the gut microbiome and metabolome [61].

It has been proposed that GTE’s health benefits may be linked to the effects catechins exert on particular bacterial species in the gut. For instance, catechins have been shown to affect the growth of *Bacteroidetes* and *Firmicutes* [62]. It is especially important to note that the relative proportion of *Bacteroidetes* to *Firmicutes* and bacterial alpha diversity are markedly decreased in both obese humans and obese mice [25,63,64,65]. Further studies have confirmed EGCG-induced changes to gut ecology [66]. Additionally, administration of green tea polyphenols appears to modulate gut microbiota diversity, including restoration of the *Bacteroidetes* to *Firmicutes* ratio resulting in body weight loss in mice fed a high fat diet [65]. Interestingly, another recent study that used liquid green tea reported opposite results with a decrease observed in the *Bacteroidetes* to *Firmicutes* ratio [42]. In our study, coincident with an increased *Bacteroides* to *Firmicutes* ratio, we also found an increase in *A. muciniphila*, a mucin degrading bacteria, which has been reported as a beneficial gut microbe associated with body fat reduction, correction of dyslipidemia and reduced insulin resistance [67].

In conclusion, we demonstrate that dGTE, when administered to non-fasting and genetically uncompromised mice, does not elicit hepatotoxic effects even when administered at doses as high as 659 mg/kg bw/day. Additional studies, however, will be needed to delineate the role of other confounding factors like caffeine, which may decrease tolerance to GTE. We further demonstrate that dGTE doses ~200 mg/kg bw can substantially modulate the gut microbiome, leading to increases in the health-beneficial bacteria *Akkermansia sp*. These findings may give insight into the potential weight management properties of GTE; however, future studies are needed to fully delineate this effect.

## Figures and Tables

**Table 1:** Phytochemical characterization of decaffeinated green tea extract (dGTE) product used in the study.

Ingredient	mg/Capsule
Caffeine	9.9
Gallocatechin	4.5
Epigallocatechin	17.2
Catechin	2.6
Epicatechin	14.4
Epigallocatechin gallate	180.3
Gallocatechin gallate	4.5
Epicatechin gallate	31.1
Catechin gallate	0.6
Sum of catechins	255.3
Sum of E-catechins	243.0

**Table 2:** Clinical chemistry parameters after dosing with dGTE for 24 h and 2 weeks.

**24 h**	**Units**	**Vehicle**	**1X**	**3X**	**10X**
Total Bilirubin	mg/dL	<0.2	<0.2	<0.2	<0.2
ALT	U/L	28.8 (±8.8)	52.6 (±20.2)	34.4 (±14.8)	32.2 (±11.9)
AST	U/L	66.2 (±5.7)	84.2 (±8.2)	65.2 (±9.8)	68.2 (±10.3)
GGT	U/L	<3.0	<3.0	<3.0	<3.0
Alkaline Phosphatase	IU/L	197 (±3.8)	196.2 (±7.3)	163.8 (±13.5) *	189.8 (±6.4)
**2 weeks**	**Units**	**Vehicle**	**1X**	**3X**	**10X**
Total Bilirubin	mg/dL	<0.2	<0.2	0.2	<0.2
ALT	U/L	28.8 (±8.8)	24.8 (±2.3)	34.2 (±14.4)	33.5 (±11.6)
AST	U/L	66.2 (±5.7)	49.2 (±12.9)	70.8 (±13.9)	67.6 (±10.4)
GGT	U/L	<3.0	4.8 (±1.3)	<3.0	<3.0
Alkaline Phosphatase	IU/L	148 (±4.5)	91.6 (±12.9) **	131.2 (±18.1)	140.2 (±6.7)

Data presented as mean +/- SEM (*n* = 5 per group) * *p* < 0.05, ** *p* < 0.01 compared to vehicle.

## Supplementary Materials

The following are available online at https://www.mdpi.com/2072-6643/11/4/776/s1, Figure S1: Analysis of dGTE acute toxicity. Heart-to-body weight ratio (A) and kidney-to-body weight ratio (B). * *p* < 0.05, Figure S2: Analysis of dGTE sub-acute toxicity. Heart-to-body weight ratio (A) and kidney-to-body weight ratio (B). * *p* < 0.05, Figure S3: High abundance taxa in individual gut microbiome samples, Table S1: Taqman Custom Array targets, Table S2: Listing of taxa abundance.
