# Supplementary material for: Decaffeinated Green Tea Extract Does Not Elicit Hepatotoxic Effects and Modulates the Gut Microbiome in Lean B6C3F1 Mice
Source: Nutrients. 2019 Apr 3;11(4):776. doi: 10.3390/nu11040776 (PMC6521095; doi:10.3390/nu11040776)
Supplement: Supplementary file 1 [file nutrients-11-00776-s001.zip › Table S1.docx]

| Supplementary Table 1. Taqman Custom Array Targets | | | |
| --- | --- | --- | --- |
| Target Name | Probe | Target Name | Probe |
| 18S | Hs99999901_s1 | Ipo8 | Mm01255158_m1 |
| Abcb11 | Mm00445168_m1 | Krt18 | Mm01601704_g1 |
| Abcb1a | Mm00440761_m1 | Krt8 | Mm04209403_g1 |
| Abcb4 | Mm00435630_m1 | L2hgdh | Mm00778242_s1 |
| Abcc2 | Mm00496899_m1 | Lgr5 | Mm00438890_m1 |
| Abcc3 | Mm00551550_m1 | Lpl | Mm00434764_m1 |
| Actb | Mm00607939_s1 | Lss | Mm00461312_m1 |
| Aldoa | Mm00833172_g1 | Maob | Mm00555412_m1 |
| Asah1 | Mm00480021_m1 | Map3k6 | Mm00522235_m1 |
| Atp8b1 | Mm01257688_m1 | Mbl2 | Mm00487623_m1 |
| Avpr1a | Mm00444092_m1 | Mcm10 | Mm00712529_m1 |
| B2m | Mm00437762_m1 | Mlxipl | Mm02342723_m1 |
| Bhmt | Mm04210521_g1 | Mrps18b | Mm00458794_m1 |
| Btg2 | Mm00476162_m1 | Nqo1 | Mm01253561_m1 |
| Car3 | Mm01281795_m1 | Nus1 | Mm01189641_m1 |
| Casp3 | Mm01195085_m1 | Osmr | Mm01307326_m1 |
| Ccng1 | Mm00438084_m1 | Pdyn | Mm00457573_m1 |
| Cd36 | Mm01135198_m1 | Pgk1 | Mm00435617_m1 |
| Cd68 | Mm03047340_m1 | Pla2g12a | Mm00458226_m1 |
| Cdc14b | Mm00553630_m1 | Polr2a | Mm00839493_m1 |
| Cdkn1a | Mm04205640_g1 | Ppara | Mm00440939_m1 |
| Col4a1 | Mm01210125_m1 | Ppia | Mm02342430_g1 |
| Cryl1 | Mm00504138_m1 | Psme3 | Mm00839833_m1 |
| Cxcl12 | Mm00445553_m1 | Pygl | Mm01289790_m1 |
| Cyp1a2 | Mm00487224_m1 | Rb1 | Mm00485586_m1 |
| Ddit4l | Mm00513313_m1 | Rdx | Mm01177363_m1 |
| Ddx39 | Mm00677594_g1 | Rhbg | Mm00491234_m1 |
| Dnajb11 | Mm00518196_m1 | Rplp2 | Mm00782638_s1 |
| Dnajc3 | Mm00515299_m1 | S100a8 | Mm00496696_g1 |
| Emc9 | Mm01313863_m1 | Scd1 | Mm00772290_m1 |
| Fabp1 | Mm00444340_m1 | Serpina3n | Mm00776439_m1 |
| Fads1 | Mm00507605_m1 | Serpine1 | Mm00435860_m1 |
| Fasn | Mm00662319_m1 | Skil | Mm00456917_m1 |
| Fmo1 | Mm00515795_m1 | Slc17a3 | Mm00506321_m1 |
| Gapdh | Mm99999915_g1 | Slc2a3 | Mm00441483_m1 |
| Gclc | Mm00802655_m1 | Slc39a6 | Mm00507295_m1 |
| Gsr | Mm00439154_m1 | Slc51a | Mm00521530_m1 |
| Gusb | Mm00446953_m1 | Srebf1 | Mm00550338_m1 |
| Hao2 | Mm00469507_m1 | Tagln | Mm00441661_g1 |
| Hmbs | Mm00660262_g1 | Tbp | Mm00446973_m1 |
| Hmox1 | Mm00516005_m1 | Tfrc | Mm00441941_m1 |
| Hpn | Mm01152654_m1 | Thrsp | Mm01273967_m1 |
| Hprt | Mm00446968_m1 | Timm10b | Mm00727252_s1 |
| Hyou1 | Mm00491279_m1 | Tmem2 | Mm00459599_m1 |
| Icam1 | Mm00516023_m1 | Txnrd1 | Mm00443675_m1 |
| Igfals | Mm01962637_s1 | Ubc | Mm01201237_m1 |
| Il6st | Mm00439665_m1 | Wipi1 | Mm00461219_m1 |
| Ipo4 | Mm00502820_m1 | Ywhaz | Mm01158417_g1 |
