# Supplementary figures and images for: Decaffeinated Green Tea Extract Does Not Elicit Hepatotoxic Effects and Modulates the Gut Microbiome in Lean B6C3F1 Mice
Source: Nutrients. 2019 Apr 3;11(4):776. doi: 10.3390/nu11040776 (PMC6521095; doi:10.3390/nu11040776)

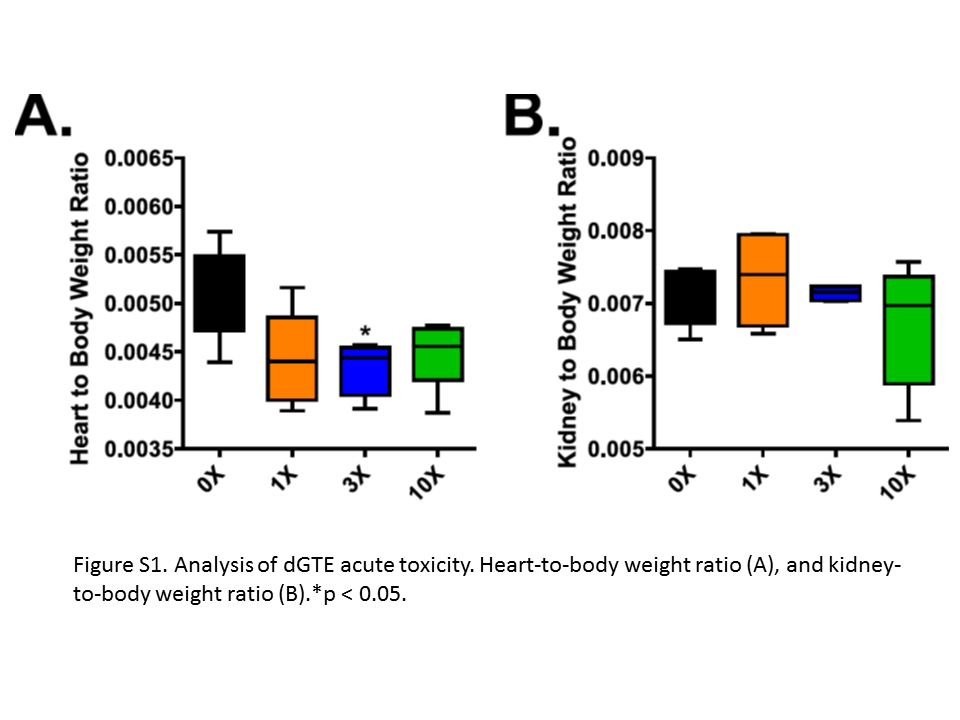

Supplement: Supplementary file 1 [file nutrients-11-00776-s001.zip › Figure S1.tif]

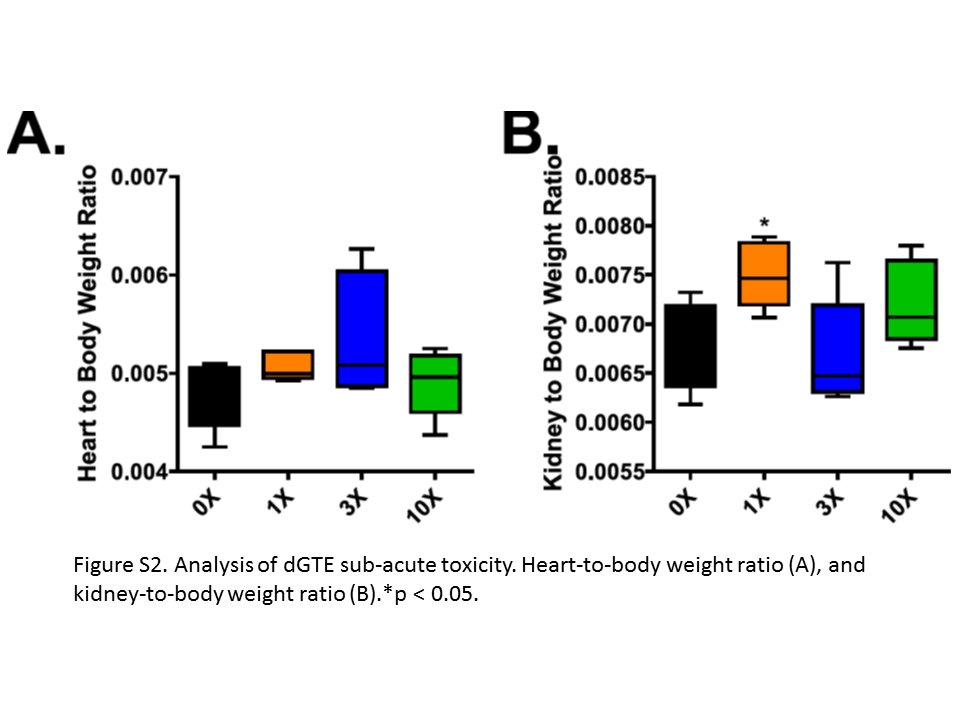

Supplement: Supplementary file 1 [file nutrients-11-00776-s001.zip › Figure S2.tif]

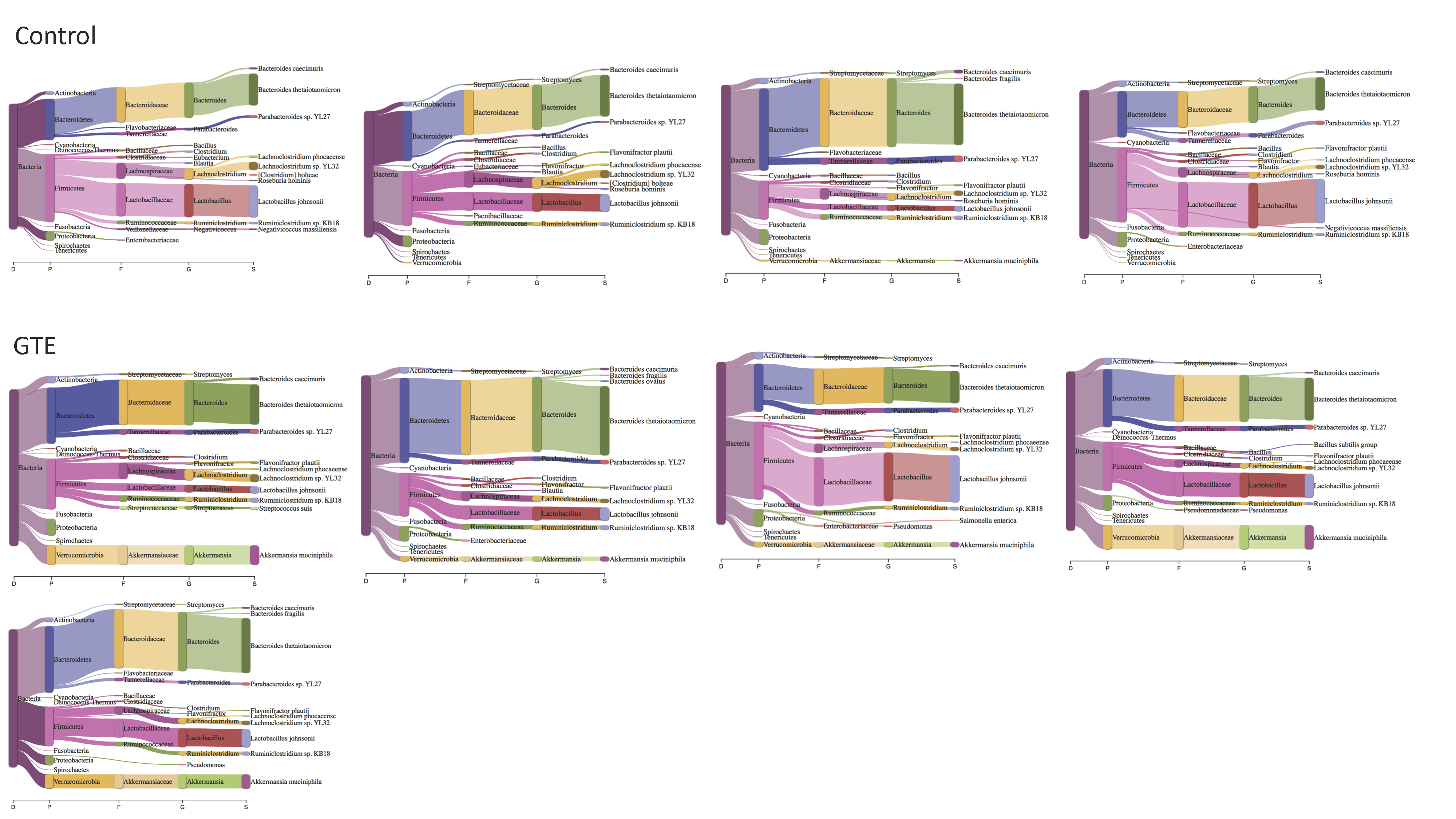

Supplement: Supplementary file 1 [file nutrients-11-00776-s001.zip › Figure S3.tiff]
